# Effectiveness of Immunosuppressive Therapy in Preventing the Progression of Ocular Myasthenia Gravis to Generalized Myasthenia Gravis: A Systematic Review and Meta-Analysis

**DOI:** 10.7759/cureus.80172

**Published:** 2025-03-06

**Authors:** Scott Nall, Jawaria Majeed, Abdulazizi Ahmed Mohsen Alrashidi, Mohammad Alhneif, Abdul Hannan Asghar, Abdullah Mubarik, Ibrahim Ahmed Mohsen Alrashidi, Danish Allahwala

**Affiliations:** 1 Medicine, Central Michigan University (CMU) School of Medicine, Mount Pleasant, USA; 2 Gastroenterology and Hepatology, Nishtar Medical University, Multan, PAK; 3 Health Affairs, Ministry of National Guard, Riyadh, SAU; 4 Neuropathology, University of Texas Health Science Center at San Antonio, San Antonio, USA; 5 Medicine, Quaid-e-Azam Institute of Nursing and Allied Health Sciences, Lahore, PAK; 6 Pharmacy, Al Nakheel Medical Centre, Muqaba, BHR; 7 Nephrology, Fatima Memorial Hospital, Lahore, PAK

**Keywords:** disease progression, generalized myasthenia gravis, immunosuppressive therapy, meta-analysis, ocular myasthenia gravis

## Abstract

Ocular myasthenia gravis (OMG) is a localized form of myasthenia gravis (MG) that primarily affects the extraocular muscles, causing symptoms such as ptosis and diplopia. Without treatment, patients with OMG can progress to generalized MG (gMG), which involves systemic muscle weakness and can lead to severe complications. Immunosuppressive therapy aims to modulate the autoimmune response that underlies MG by reducing the production of pathogenic autoantibodies against the acetylcholine receptors (AChRs) or related neuromuscular junction components. This systematic review and meta-analysis evaluated the effectiveness of immunosuppressive therapy in preventing progression from OMG to gMG. A comprehensive literature search was conducted across PubMed, Web of Science, and Embase databases for studies published through January 2025. Ten retrospective observational studies met inclusion criteria, comprising 1,458 participants, with 761 receiving immunosuppression therapy. The meta-analysis revealed that immunosuppressive therapy significantly reduced the risk of generalization compared to control groups (OR: 0.23, 95%CI: 0.15-0.38, I^2^: 34%), representing a 77% decrease in progression rate. Sensitivity analyses demonstrated robust findings, with consistent ORs ranging from 0.21 to 0.27 after sequential removal of individual studies. The funnel plot showed no significant publication bias. However, the review was limited by the retrospective nature of included studies, moderate heterogeneity, and predominant focus on Western populations. These findings support the use of immunosuppressive therapy in preventing OMG progression, though further research through multicenter randomized controlled trials is needed to establish optimal treatment protocols, particularly in Asian populations. Future studies should also investigate biomarkers for treatment response and compare effectiveness between different immunosuppressive regimens.

## Introduction and background

Ocular myasthenia gravis (OMG) is a subset of myasthenia gravis (MG), a neuromuscular disorder characterized by autoimmune-mediated disruption of the neuromuscular junction [1]. OMG accounts for approximately 50-60% of MG cases at onset, with nearly half of these patients progressing to generalized MG (gMG) within two years [2]. Patients with OMG present with isolated ocular symptoms, including ptosis and diplopia, caused by the weakness of extraocular muscles [1]. Although OMG is generally considered a more localized form of the disease, it is well-documented that a significant proportion of patients progress to gMG within a few years [3]. This progression, which affects not only ocular but also bulbar and limb muscles, is associated with more severe symptoms, a higher risk of respiratory involvement, and increased healthcare resource utilization [4]. The transition from OMG to gMG occurs in approximately 60-80% of patients within the first two years of diagnosis, highlighting the need for effective strategies to prevent this generalization [5]. 

The management of OMG primarily focuses on symptom control, and immunosuppressive therapies have become a cornerstone of treatment. These therapies, which include corticosteroids, azathioprine, mycophenolate mofetil, and tacrolimus, are designed to modulate the immune response and reduce the production of antibodies that impair neuromuscular transmission [6,7]. While the efficacy of these therapies in improving ocular symptoms has been established, their role in preventing disease progression remains unclear. Although some studies suggest that early use of immunosuppressants may reduce the risk of progression to gMG, other research has failed to show significant differences compared to symptomatic treatment alone [8]. The lack of consensus on this issue complicates clinical decision-making and underscores the importance of synthesizing available evidence to guide treatment strategies. 

Several factors may influence the progression of OMG to gMG, including the severity of ocular symptoms at diagnosis, the presence of acetylcholine receptor antibodies, and the age of onset [9]. Additionally, the timing, type, and duration of immunosuppressive therapy may also affect the likelihood of progression. Despite these variables, no large-scale studies have definitively established the optimal approach to preventing generalization in OMG patients [10]. Given the variability in treatment responses and the evolving landscape of immunosuppressive options, it seemed important to critically assess the collective evidence through a systematic review and meta-analysis. 

This study aims to systematically review the available literature on the use of immunosuppressive therapies in OMG and evaluate their effectiveness in preventing the progression to gMG. By pooling data from relevant studies, we hope to clarify the role of immunosuppressants in disease modification and provide evidence-based recommendations to inform clinical practice. Ultimately, this analysis will help improve the management of OMG and potentially alter the course of the disease for many patients at risk of generalization. 

## Review

Methodology 

Literature Search 

A comprehensive and systematic literature search was conducted to identify relevant studies assessing the impact of immunosuppressant therapies on preventing the progression from OMG to gMG. The search included studies published in English from inception to January 15, 2025. Databases including PubMed, Web of Science, and Embase were queried using a combination of MeSH terms and keywords related to "ocular myasthenia gravis", "immunosuppressant therapies", "generalization", and "disease progression". The search strategy also included terms for specific immunosuppressive agents such as corticosteroids, azathioprine, mycophenolate mofetil, and tacrolimus. A search was also performed in Google Scholar to avoid any article being missed. Additionally, the reference lists of included articles and relevant reviews were hand-searched to ensure no pertinent studies were missed. The search was performed by two authors (AA and MA) independently. Any disagreement between two authors was resolved through discussion or involvement of a third author (SN) if required. 

*Study Selection* 

Studies were included in the review if they met the following criteria: (1) patients diagnosed with OMG, (2) investigation of immunosuppressant therapies to prevent the progression to gMG, (3) clinical studies including randomized controlled trials (RCTs), cohort studies, and case-control studies, and (4) available data on the rate of generalization to gMG. Exclusion criteria were studies that: (1) did not assess the primary outcome of generalization to gMG, (2) focused solely on non-immunosuppressive treatments, and (3) were review articles, case reports, editorials, and animal studies. Two independent reviewers (JM and IA) screened the titles and abstracts of all records identified in the search. Full-text articles were reviewed for eligibility, and any disagreements were resolved through consensus or discussion with a third reviewer (SN). 

Quality Assessment 

The quality of the included studies was assessed using appropriate tools depending on the study design. For RCTs, the Cochrane Risk of Bias tool (The Cochrane Collaboration, London, United Kingdom) was employed, which assesses risk across domains such as selection bias, performance bias, detection bias, and attrition bias. For cohort and case-control studies, the Newcastle-Ottawa Scale (NOS), an ongoing collaboration between the Universities of Newcastle, Australia, and Ottawa, Canada, was used to evaluate the selection of study participants, comparability of groups, and outcome assessment. Studies were rated as low, moderate, or high risk of bias based on the scoring system of the respective tool. The quality assessment was performed independently by two reviewers, and any discrepancies were resolved by discussion. 

Data Extraction 

Data were independently extracted from the included studies by two reviewers (AA and AM) using a standardized data extraction form. The following information was extracted: first author, year of publication, study design, sample size, patient characteristics (e.g., age, gender, duration of OMG), details of the immunosuppressive therapies used, and the reported outcomes regarding the progression to gMG. The primary outcome was the rate of progression from OMG to gMG, defined as the clinical diagnosis of gMG during follow-up. 

Statistical Analysis 

The primary outcome of this systematic review and meta-analysis was the rate of generalization to gMG in patients with OMG treated with immunosuppressive therapies. Pooled estimates of the risk of progression were calculated using random-effects models, as we expected some heterogeneity across studies due to differences in study populations and treatments. The odds ratio (OR) with a 95% confidence interval (CI) was used to compare the likelihood of progression to gMG between patients receiving immunosuppressive therapy and those not receiving such treatment. Heterogeneity among studies was assessed using the I² statistic, and potential sources of heterogeneity were explored through subgroup analysis based on factors such as the type of immunosuppressant used and study design. Sensitivity analysis was conducted to examine the robustness of the results by excluding studies with a high risk of bias. All statistical analyses were performed using RevMan Version 5.4.1 (The Cochrane Collaboration) and a p-value of less than 0.05 was considered statistically significant. 

Results 

**Figure 1:**
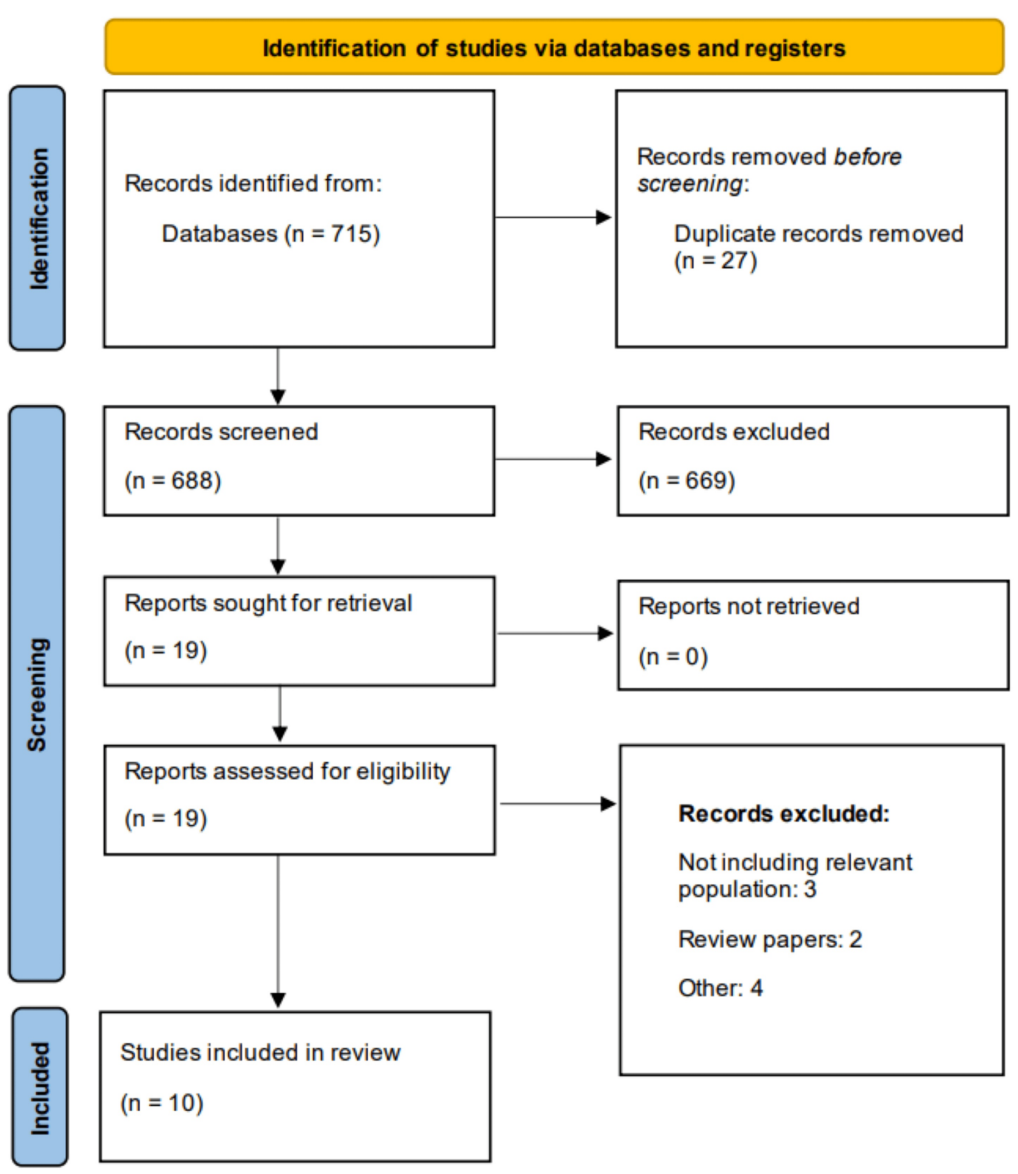
PRISMA flowchart (study selection process) PRISMA: Preferred Reporting Items for Systematic Reviews and Meta-Analyses

The online systematic search yielded 715 studies. After removing duplicates, 688 studies were initially screened. Full-text screening of 19 studies was done based on inclusion and exclusion criteria. In the end, 10 studies were selected to be part of this meta-analysis comprising a total of 1458 participants, of which 761 subjects received immunosuppression therapy. Figure 1 shows the Preferred Reporting Items for Systematic Reviews and Meta-Analyses (PRISMA) flowchart of study selection. Table 1 presents the characteristics of the included studies. The publication year of the included studies ranged from 2003 to 2024. The majority of the studies were carried out in the United States (n=6). All included studies were retrospective observational. The mean age of the included studies ranged from 48 to 75.9 years. Follow-up of included studies ranged from two years to 10 years. Table 2 presents the quality assessment of the included studies.

**Table 1:** Characteristics of included studies

Author	Year of publication	Study Design	Region	Groups	Sample Size	Mean age	Number of male participants	Follow-up duration
Allen et al. [11]	2009	Retrospective	United States	Immunosuppression	15	75.9	24	4.5 Years
				Control	24			
Bhanushalli et al. [12]	2008	Retrospective	United States	Immunosuppression	27	52	19	3 Years
				Control	8			
Kupersmith et al. [13]	2003	Retrospective	United States	immunosuppression	58	48	57	2 Years
				Control	36			
Kupersmith [14]	2009	Retrospective	United States	Immunosuppression	55	54	55	7 Years
				Control	32			
Mee et al. [15]	2003	Retrospective	Australia	Immunosuppression	12	55.1	19	10 Years
				Control	22			
Menon et al. [16]	2024	Retrospective	Canada	Immunosuppression	49	58.8	44	7 Years
				Control	49			
Monsul et al. [17]	2004	Retrospective	United States	Immunosuppression	27	53	31	2 Years
				Control	29			
Nagia et al. [18]	2015	Retrospective	United States	Immunosuppression	76	61.5	106	2 Years
				Control	82			
Ruan et al. [19]	2021	Retrospective	China	Immunosuppression	425	49	443	2 Years
				Control	388			
Zach et al. [20]	2013	Retrospective	Austria	Immunosuppression	17	54	24	2 Years
				Control	27			

**Table 2:** Quality assessment of included studies

Study details	Selection	Comparability	Assessment	Overall
Allen et al., 2009 [11]	4	2	2	Good
Bhanushalli et al., 2008 [12]	3	2	3	Good
Kupersmith et al., 2003 [13]	4	2	2	Good
Kupersmith, 2009 [14]	4	1	3	Good
Mee et al., 2003 [15]	3	2	3	Good
Menon et al., 2024 [16]	3	2	3	Good
Monsul et al., 2004 [17]	2	2	2	Fair
Nagia et al., 2015 [18]	3	1	2	Fair
Ruan et al., 2021 [19]	3	1	3	Good
Zach et al., 2013 [20]	4	2	3	Good

Effect of Immunosuppressant on the Generalization Rate 

**Figure 2:**
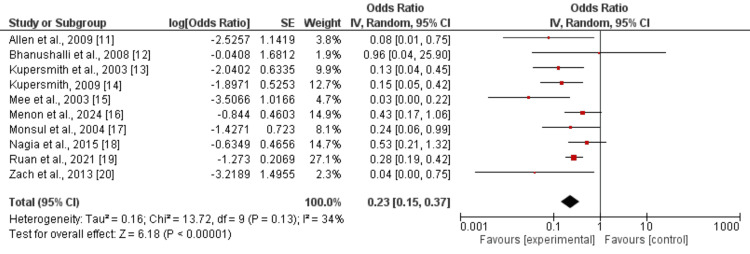
Effect of immunosuppressants on generalization References: [11-20]

A comparison of the effect of immunosuppression treatment and control on the rate of generalization is presented in Figure 2. The odds of developing generalization were significantly lower in patients receiving immunosuppressants compared to control groups (OR: 0.23, 95%CI: 0.15 to 0.38, I^2^: 34%), showing that immunosuppressant therapy decreased the generalization rate by 77%. 

Sensitivity Analysis and Publication Bias 

**Figure 3:**
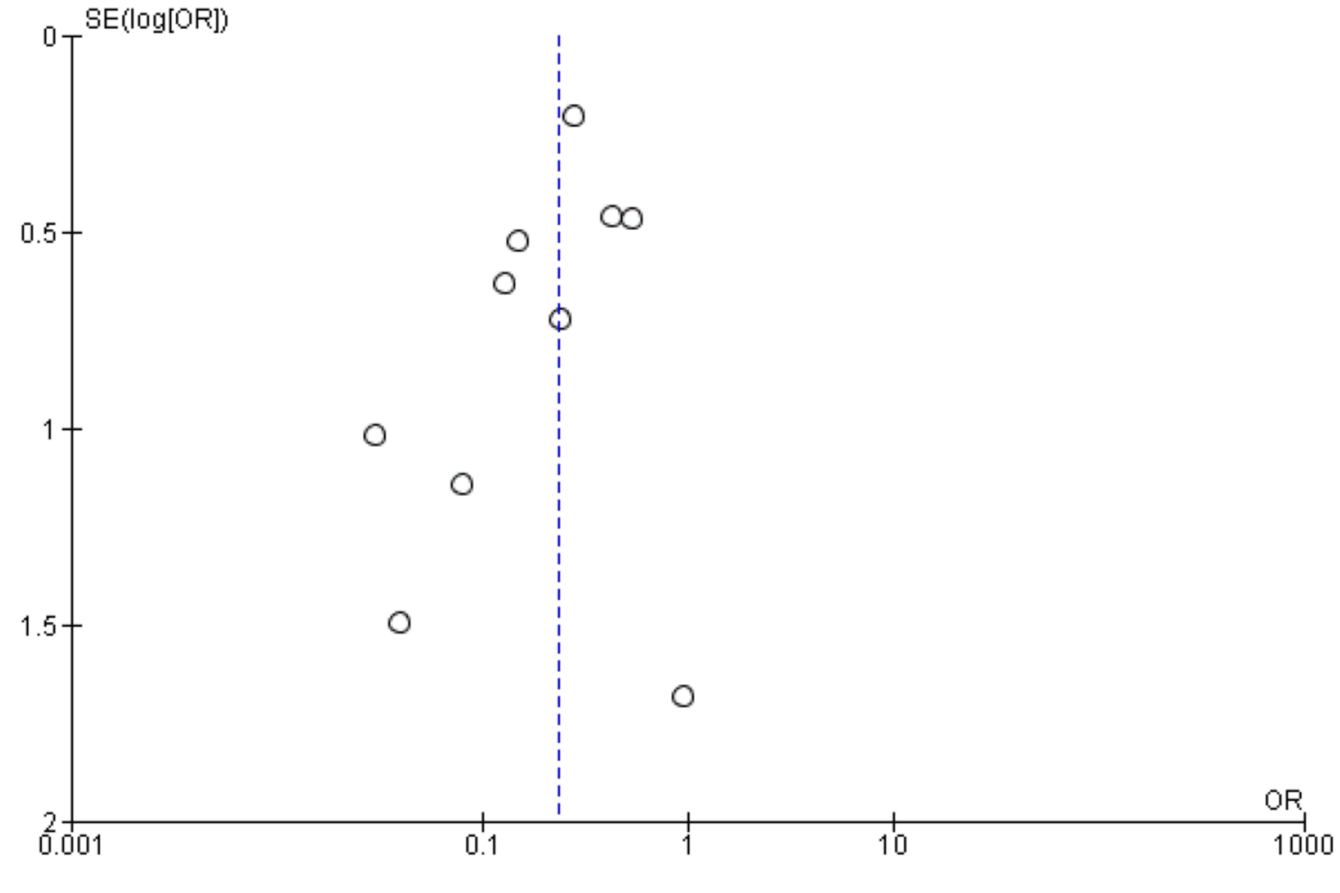
Funnel plot (showing publication bias)

A sensitivity analysis was conducted to assess the robustness of the findings comparing immunosuppressant therapy to control for the outcome of generalization, and the findings are represented in Table 3. Each study was sequentially removed, and the OR remained consistent, ranging from 0.21 to 0.27, with CIs overlapping. The sensitivity analysis revealed that removing Mee et al.'s study reduced heterogeneity to 12%, suggesting it was a key contributor to variability [15]. Despite this, the OR remained stable, ranging from 0.21 to 0.27, with overlapping CIs, indicating a consistent treatment effect of immunosuppressant therapy versus control on generalization. This suggests that while Mee et al.'s study [15] contributed to heterogeneity, its exclusion did not significantly alter the overall conclusions, reinforcing the robustness of the findings. The funnel plot shown in Figure 3 appears to be relatively symmetrical, with studies distributed evenly around the central effect estimate. There is no clear evidence of asymmetry, which suggests that publication bias is unlikely. Additionally, the presence of studies with larger standard errors (smaller sample sizes) on both sides of the plot further supports the absence of significant publication bias. 

**Table 3:** Sensitivity analysis (removing one study at a time) OR: Odds ratio; CI: Confidence interval

Study details	OR (95% CI)	I^2^
Allen et al., 2009 [11]	0.25 (0.14, 0.39)	37%
Bhanushalli et al., 2008 [12]	0.22 (0.14, 0.36)	39%
Kupersmith et al., 2003 [13]	0.25 (0.15, 0.41)	36%
Kupersmith, 2009 [14]	0.25 (0.15, 0.41)	36%
Mee et al., 2003 [15]	0.27 (0.19, 0.39)	12%
Menon et al., 2024 [16]	0.21 (0.12, 0.35)	35%
Monsul et al., 2004 [17]	0.23 (0.14, 0.38)	42%
Nagia et al., 2015 [18]	0.21 (0.13, 0.33)	28%
Ruan et al., 2021 [19]	0.21 (0.11, 0.38)	41%
Zach et al., 2013 [20]	0.24 (0.16, 0.39)	34%

Discussion 

The aim of this systematic review and meta-analysis was to evaluate the effect of immunosuppressant therapy on the risk of secondary generalization in patients with OMG. The findings suggest that immunosuppressants can significantly reduce this risk. A meta-analysis by Li et al., which included eight studies, reported similar results [21]. Moderate heterogeneity was observed in our study, prompting a sensitivity analysis to identify its source. The analysis revealed that Mee et al.'s study contributed the most to heterogeneity, likely due to its longer follow-up period. 

Immunosuppressants may prevent secondary generalization in OMG by modulating the autoimmune response at multiple levels. They help reduce the production of pathogenic autoantibodies, particularly those targeting the acetylcholine receptors (AChRs) at the neuromuscular junction, thereby limiting the immune-mediated attack on muscle function [22]. Additionally, these agents suppress T-cell activation and cytokine release, dampening the inflammatory cascade that contributes to disease progression. By stabilizing neuromuscular transmission and preventing widespread synaptic dysfunction, immunosuppressants may slow or halt the transition from OMG to gMG, thereby preserving muscle function and reducing symptom burden [23]. Although the autoimmune nature of MG is well established, evidence supporting the protective role of immunosuppressive therapy in OMG remains limited. This gap in research is primarily due to challenges in conducting controlled studies within the OMG population. For instance, a past randomized clinical trial designed to evaluate the effectiveness of prednisone in OMG had to be discontinued prematurely due to enrollment difficulties, ultimately including only 11 participants [24]. 

The optimal duration of immunosuppression for managing OMG remains unclear, with treatment duration varying across studies. In a study by Sommer et al., patients received prednisolone for an average of 32.3 months, while azathioprine therapy lasted approximately 43.7 months [25]. Similarly, Zach et al. recommended a minimum treatment duration of three months with prednisolone [20]. However, long-term immunosuppression must be carefully balanced against potential adverse effects, including infection risk, metabolic complications, and drug-specific toxicities [16]. Further research is needed to establish standardized treatment duration guidelines, optimize tapering strategies, and determine the most effective maintenance regimens for preventing disease progression while minimizing side effects. Large-scale, multicenter RCTs are needed to confirm the protective role of immunosuppression in preventing generalization. Additionally, studies exploring biomarkers that predict treatment response could help in patient stratification and individualized therapy. Comparative effectiveness research between different immunosuppressants and combination regimens may provide further insights. Long-term follow-up studies are also essential to assess relapse rates and the feasibility of tapering or discontinuing therapy in controlled disease states, ultimately guiding clinical decision-making. 

This meta-analysis has several limitations. First, all included studies were conducted in developed countries, primarily in the United States. There is a notable lack of research from Asian, African, and other populations, limiting the generalizability of findings, as previous studies have suggested a lower generalization rate in OMG patients from these regions [26]. Second, moderate heterogeneity was observed across studies, but subgroup analysis could not be performed due to insufficient data. Lastly, all included studies were retrospective observational in design, inherently prone to selection bias and confounding. To validate the role of immunosuppressants, particularly in mild cases and at low doses, well-designed prospective cohort studies and multicenter RCTs are essential. 

## Conclusions

This meta-analysis demonstrates that immunosuppressive therapy significantly reduces the risk of generalization from OMG to gMG, with a steep decrease in progression rate compared to controls. The findings were robust across sensitivity analyses, with minimal evidence of publication bias. However, the review's limitations include a reliance on retrospective studies primarily from Western populations and moderate heterogeneity across studies. Future research should focus on conducting multicenter RCTs, particularly in Asian populations, to establish optimal treatment protocols and duration. Additionally, studies exploring biomarkers for treatment response and comparative effectiveness between different immunosuppressive regimens are needed. Despite these limitations, the evidence supports the use of immunosuppressive therapy in preventing disease progression in OMG patients.

## References

[1] Behbehani R (2023). Ocular myasthenia gravis: a current overview. Eye Brain.

[2] Shuey NH (2022). Ocular myasthenia gravis: a review and practical guide for clinicians. Clin Exp Optom.

[3] Narita T, Nakane S, Nagaishi A (2023). Immunotherapy for ocular myasthenia gravis: an observational study in Japan. Ther Adv Neurol Disord.

[4] De Bleecker JL, Remiche G, Alonso-Jiménez A, Van Parys V, Bissay V, Delstanche S, Claeys KG (2024). Recommendations for the management of myasthenia gravis in Belgium. Acta Neurol Belg.

[5] Guo RJ, Gao T, Ruan Z (2022). Risk factors for generalization in patients with ocular myasthenia gravis: a multicenter retrospective cohort study. Neurol Ther.

[6] Morren J, Li Y (2020). Maintenance immunosuppression in myasthenia gravis, an update. J Neurol Sci.

[7] García Estévez DA, Pardo Fernández J (2023). Myasthenia gravis. Update on diagnosis and therapy. Med Clin (Barc).

[8] Hehir MK 2nd, Li Y (2022). Diagnosis and management of myasthenia gravis. Continuum (Minneap Minn).

[9] Mazzoli M, Ariatti A, Valzania F, Kaleci S, Tondelli M, Nichelli PF, Galassi G (2018). Factors affecting outcome in ocular myasthenia gravis. Int J Neurosci.

[10] Kemchoknatee P, Arepagorn A, Srisombut T (2021). Ocular manifestation and generalization after ocular onset in ocular myasthenia gravis: a 5-year analysis. Asian Pac J Allergy Immunol.

[11] Allen JA, Scala S, Jones HR (2010). Ocular myasthenia gravis in a senior population: diagnosis, therapy, and prognosis. Muscle Nerve.

[12] Bhanushali MJ, Wuu J, Benatar M (2008). Treatment of ocular symptoms in myasthenia gravis. Neurology.

[13] Kupersmith MJ, Latkany R, Homel P (2003). Development of generalized disease at 2 years in patients with ocular myasthenia gravis. Arch Neurol.

[14] Kupersmith MJ (2009). Ocular myasthenia gravis: treatment successes and failures in patients with long-term follow-up. J Neurol.

[15] Mee J, Paine M, Byrne E, King J, Reardon K, O'Day J (2003). Immunotherapy of ocular myasthenia gravis reduces conversion to generalized myasthenia gravis. J Neuroophthalmol.

[16] Menon D, Alharbi M, Katzberg HD, Bril V, Mendoza MG, Barnett-Tapia C (2024). Effect of immunosuppression in risk of developing generalized symptoms in ocular myasthenia gravis: a retrospective cohort study. Neurology.

[17] Monsul NT, Patwa HS, Knorr AM, Lesser RL, Goldstein JM (2004). The effect of prednisone on the progression from ocular to generalized myasthenia gravis. J Neurol Sci.

[18] Nagia L, Lemos J, Abusamra K, Cornblath WT, Eggenberger ER (2015). Prognosis of ocular myasthenia gravis: retrospective multicenter analysis. Ophthalmology.

[19] Ruan Z, Guo R, Zhou H (2022). Association of immunosuppression treatment with generalization among patients with ocular myasthenia gravis: a propensity score analysis. Eur J Neurol.

[20] Zach H, Cetin H, Hilger E (2013). The effect of early prednisolone treatment on the generalization rate in ocular myasthenia gravis. Eur J Neurol.

[21] Li M, Ge F, Guo R (2019). Do early prednisolone and other immunosuppressant therapies prevent generalization in ocular myasthenia gravis in Western populations: a systematic review and meta-analysis. Ther Adv Neurol Disord.

[22] Koneczny I, Herbst R (2019). Myasthenia gravis: pathogenic effects of autoantibodies on neuromuscular architecture. Cells.

[23] Dresser L, Wlodarski R, Rezania K, Soliven B (2021). Myasthenia gravis: epidemiology, pathophysiology and clinical manifestations. J Clin Med.

[24] Benatar M, Mcdermott MP, Sanders DB (2016). Efficacy of prednisone for the treatment of ocular myasthenia (EPITOME): a randomized, controlled trial. Muscle Nerve.

[25] Evoli A, Tonali P, Bartoccioni E, Lo Monaco M (1988). Ocular myasthenia: diagnostic and therapeutic problems. Acta Neurol Scand.

[26] Gui M, Luo X, Lin J (2015). Long-term outcome of 424 childhood-onset myasthenia gravis patients. J Neurol.

